# Quantification of human polyomaviruses MCPyV and HPyV6 in malignant and non-malignant skin lesions^☆^

**DOI:** 10.1016/j.abd.2022.02.006

**Published:** 2023-01-10

**Authors:** Marianna T. Venceslau, Gabriella R.M. da Costa, Maria Angelica A.M. Guimarães, Rafael B. Varella, Flavio B. Luz

**Affiliations:** a Department of Preventive Medicine, Hospital Universitário Clementino Fraga Filho, Universidade Federal do Rio de Janeiro, Rio de Janeiro, RJ, Brazil; b Department of Microbiology and Parasitology, Biomedical Institute, Universidade Federal Fluminense, Niteroi, RJ,Brazil; c Department of Clinical Medicine, Sector of Dermatology, Hospital Universitário Antônio Pedro, Universidade Federal Fluminense, Niteroi, RJ, Brazil

**Keywords:** Carcinogenesis, Merkel cell polyomavirus, Polyomavirus, Skin neoplasms, Virus diseases

## Abstract

**Background:**

Human Polyomaviruses such as MCPyV and HPyV6 are frequently found as part of healthy skin microbiota and have been associated with Merkel cell carcinoma (MCC), pruritic and dyskeratotic dermatoses, respectively. Their presence in other types of skin conditions varies greatly depending on lesion type and population.

**Objective:**

To analyse comparatively the presence of MCPyV and HPyV6 in nonmelanoma skin cancers and healthy skin.

**Methods:**

The authors utilized qPCR techniques to quantify these pathogens in NMSC, premalignant diseases, and healthy skin of 87 patients.

**Results:**

MCPyV was detected in over 40% of samples, while HPyV6 was in 9.6%. MCPyV load was higher in squamous cell carcinomas (SCC) compared to basal cell carcinomas (BCC) (p = 0.016) and HPyV6 showed a higher percentage of infected cells in areas of low solar exposure as well as normal skin (p = 0.012). A fair agreement (kappa = 0.301) was found between MCPyV detection in lesions and their respective perilesional skin, indicating a random process of local dissemination of the virus.

**Study limitations:**

The lack of a larger sampling of different lesion types and protein expression analyses limits the correlation findings.

**Conclusion:**

This is the first report of HPyV6 detection in the healthy skin of a Brazilian population, but the role of both polyomaviruses in NMSC has yet to be demonstrated.

## Introduction

Several viruses have been correlated to varying degrees with dermatological conditions, from exanthems and rashes to skin cancers. Among such viruses, two polyomaviruses are worth noting, Merkel Cell Polyomavirus (MCPyV) and Human Polyomavirus 6 (HPyV6). The former, also known as HPyV5, is the causal agent of Merkel Cell Carcinoma (MCC), an aggressive, lethal, and rare Non-Melanoma Skin Cancer (NMSC), mostly found in the immunocompromised and elderly.^1^ HPyV6, on the other hand, seems to also infect the skin chronically without clinical manifestation but has been associated with pruritic and dyskeratotic dermatoses.^2^ While these associations are established, both viruses have been found in different lesions, notably Non-Melanoma Skin Cancers (NMSC), as well as healthy skin and seem to constitute part of the skin flora in healthy adults.^3^

MCPyV has been investigated for involvement in the multifactorial process of other non-MCC NMSC, whether by viral persistence in the lesion or as an initial contributor to oncogenesis. Regarding HPyV6, the same questions can be made due to its epithelial tropism and expression of potentially oncogenic proteins (T-antigens). Given the tropism seen of MCPyV and HPyV6 for epithelial cells, the multifactorial nature of tumorigenesis, and known association with correlating skin diseases, the authors sought to investigate these human polyomaviruses’ DNA in hopes of elucidating possible involvements in neoplastic processes.

Expanding on a previous report, in which our group observed MCPyV in BCC via a qualitative approach, with 25.7% viral positivity,^4^ here, the authors aimed to evaluate and quantify their presence in various neoplastic and non-malignant skin lesions in a Brazilian population.

## Methodology

A total of 181 samples composed of a fresh-frozen lesion, perilesional biopsies, and normal skin derived from 87 patients treated at Antônio Pedro University Hospital (HUAP-UFF), were collected between September 2017 and December 2018. Data on age, gender, immunological status, ethnicity, and tumor location were collected during the medical examiner interview. Ethnicity, classified as ‘white’ or ‘non-white’, as defined by the dermatologist according to the patients’ phototype. While tumor location was used to infer solar exposure (high, moderate, or low).^5^ Histopathological diagnosis of all lesions was performed by the Department of Pathology of HUAP-UFF.

Regarding sampling, lesion biopsies consisted of lesion shavings, perilesional biopsies were measured by the surgical staff to ensure complete tumor removal, later confirmed ‘clear of lesional tissue’ by histopathology, and sampling of normal skin was obtained when needed to close the surgical incision. All samples were fragmented and digested with proteinase K (Promega® ‒ Madison, USA), and DNA was extracted utilizing a commercial kit following the manufacturer’s instructions (RTP® DNA/RNA Kit – Molecular Stratec Biomedical – Berlin, Germany).

MCPyV and HPyV6 DNA were quantified by TaqMan® qPCR assays based on protocols described previously.^6^, ^7^ The beta-globin gene was also amplified via qPCR to infer the percentage of infected cells ($\frac{viral\;DNA}{beta-globin\;gene/\;2}$). Statistical analysis was performed using SPSS v. 20 software (SPSS Inc. ‒ Chicago, IL, United States). Informed signed consent was obtained from all individuals who agreed to participate. This study was approved by the University’s Ethics Committee.

## Results

The demographic and clinical characteristics of the 87 NMSC patients are summarized in Table 1. Overall, the majority were classified as Caucasian (80.5%), female (55.2%), above 65 years old (58.1%), and had NMSC diagnosed at highly sun-exposed areas (70.2%), being Basal Cell Carcinoma (BCC), the most frequent (60.9%) histopathological type found. Viral frequencies according to age, gender, and ethnicity were not significant. Other lesions found, classified as premalignant, included solar elastosis, keratoacanthoma, Bowen’s disease, and hidradenitis.

**Table 1:** Patient’s characteristics (n = 87).

Variable	n (%)
Gender	
Male	39 (44.8)
Female	48 (55.2)
Ethnicity	
White	70 (80.5)
Non-white	17 (19.5)
Age (yr)	67 (17‒91)^a^
Solar exposure of lesion	
High	127 (70.2)
Moderate	30 (16.6)
Low	24 (13.3)
Skin lesion (n = 139)	
BCC	104 (76.5)
SCC	11 (8.1)
Pre-malignant	21 (15.4)

a Average (range).

MCPyV detection was elevated in overall biopsies studied (40.2%) in comparison to HPyV6 (9.6%). Although detection was statistically similar by type of lesion (Table 2), MCPyV was more frequent in neoplastic and premalignant samples altogether than in non-malignant samples (p = 0.047). MCPyV loads and percent of infected cells were not associated with the type of lesion, sample type, and solar exposure. Interestingly, the percentage of infected cells with HPyV6 was higher (p = 0.012) in low sun exposure samples and normal skin, although low sampling requires caution. MCPyV loads were higher in the SCC subtype than BCC (p = 0.016), but not in the percent of infected cells (p = 0.721) (data not shown).

**Table 2:** Frequency of detection, average viral loads, and percent of infected cells by MCPyV and HPyV6 in NMSC samples.

Variable	MCPyV			HPyV6		
	Detection (%)	Viral load	% Infected cells	Detection (%)	Viral load	% Infected cells
**Type of lesion**						
Neoplastic	16^a^	3.73 E+04	3.89 E-02	4.5	1.97 E+03	2.49 E-04
Premalignant	4.1	2.5 E+04	4.65 E-03	0	‒	‒
Non-malignant	20.1	1.74 E+04	4.21 E-02	5.1	1.91 E+03	2.32 E-01
**Solar exposure of lesion**						
High	32.5	3.24 E+04	4.88 E-02	7.3	2.00 E+03	4.6 E-04
Moderate	4.1	1.40 E+04	4.82 E-03	1.1	5.23 E+03	1.95 E-08
Low	3.6	2.99 E+03	1.24 E-02	1.1	2.59 E+03	**8.11 E-011**^b^
**Sample type**						
Lesion	18.3	1.58 E+04	4.82 E-02	5.8	2.10 E+03	2.5 E-04
Perilesional	11.2	3.73 E+04	5.54 E-02	2.9	4.72 E+02	3.9 E-04
Normal skin	10.7	6.23 E+03	3.71 E-03	1.2	2.72 E+03	**8.00 E-01**^b^

Statistically significant results (p < 0.05) are in bold letter.

a p = 0.047 with neoplastic and premalignant all together.

b p = 0.012.

The agreement of MCPyV detection between lesion and perilesional was fair (kappa = 0.301), indicating modest viral spread around the lesion (Table 3). The low detection rate of HPyV6 made this analysis inconclusive.

**Table 3:** Agreement of MCPyV detection in skin lesion and correlated perilesional tissue.

Sample type		Perilesional skin		Kappa index (95% CI)
		+	−	
Lesion	+	13	6	0.301 (0.064 to 0.538)
	−	14	26	

## Discussion

In the present study, the authors found that MCPyV detection is significantly higher in neoplastic and pre-malignant samples combined, although the viral load didn’t follow the same pattern. It is possible that inflammatory response and/or transformation processes in the injured tissue create a more permissive condition for MCPyV low-level replication, or MCPyV infection promotes a permissive state that then allows tissue injury/cellular transformation to develop. These hypotheses have primarily been posed for the development of MCPyV-positive MCC.^8^, ^9^ One of the staples of MCPyV-positive MCC is the clonal integration of viral DNA prior to or in the beginning stages of tumor-cell proliferation, which translates to a high percentage of infected cells.^1^, ^9^ This was not observed in our study, supporting the understanding that MCPyV clonal integration is relevant to MCC development but not so for other types of NMSC or pre-malignant conditions.

This is the first study to detect HPyV6 in healthy skin samples in a Brazilian population, with a previous report showing only one sample with detectable HPyV6 DNA in a Kaposi Sarcoma lesion.^10^ Reports from other population groups show a higher positivity (14%‒27.6%) of HPyV6 in healthy skin samples of immunocompetent individuals.^3^, ^11^ Taken together, these results could suggest lower overall infection in Brazilians, but certainty would require a larger population analysis. Additionally, HPyV6 has been found in 42.3% of Keratoacanthomas (KA) as well as with a high load in a single KA lesion of a melanoma patient.^12^, ^13^ In this study, however, no HPyV6 was detected in KA samples but it may be due to small sampling (n = 3).

Another note-worthy find, HPyV6 presented a higher infected cell percentage in healthy skin samples as well as in lesions with low sun exposure (p = 0.012). The viral load didn’t show varying significance. The finding of HPyV6 presence in all sample types with similar loads indicates that infection is not positively or negatively affected by tissue alterations or tumorigenic processes. The contrary can also be deduced, that HPyV6 infection seems to not further nor hinder such processes, rendering a synergistic oncogenic mechanism through mere infection improbable.

Our findings of similar viral loads independent of sample type (lesion, perilesion and normal skin) indicate viral presence is not conditioned to the presence or absence of a neoplastic lesion. This leads to a few non-mutually exclusive hypotheses: (i) Viral dispersion is independent of lesion development and the virus might be present before the beginning of lesion development; (ii) The virus disseminates to or from the lesion in a variable fashion, without partaking in the oncogenic process; (iii) Viral infection promotes a permissive state for cellular transformation and, after the onset of the oncogenic process, the virus is no longer detected in lesion due to a hit-and-run mechanism.^14^ Considering the latter, human polyomaviruses T-antigen proteins, which are known to possess oncogenic properties,^15^ could induce malignant transformation without the need for viral persistence.

## Conclusion

The findings of a higher percentage of HPyV6 infected cells in normal skin and MCPyV being more frequent in the neoplastic and premalignant groups altogether are intriguing. Overall, these results add to the body of knowledge regarding HPyV infections in different NMSC although a larger sampling could yield new correlations.

## Financial support

This study was funded by FUNADERM (Fundo de Apoio à Dermatologia). This study was also partially funded by FAPERJ (Fundação de Pesquisa do Rio de Janeiro) and Coordenação de Aperfeiçoamento de Pessoal de Nível Superior (CAPES) ‒ Financial Code 001. Varella RB. was partially supported by National Council for Scientific and Technological Development – CNPq.

## Authors’ contributions

Marianna Venceslau: Approval of the final version of the manuscript; design and planning of the study; drafting and editing of the manuscript; collection, analysis, and interpretation of data; critical review of the literature; critical review of the manuscript.

Gabriella Costa: Approval of the final version of the manuscript; collection, analysis, and interpretation of data; critical review of the literature; critical review of the manuscript.

Maria Angelica Guimarães: Approval of the final version of the manuscript; design and planning of the study; collection, analysis, and interpretation of data; effective participation in research orientation; critical review of the literature; critical review of the manuscript.

Rafael Varella: Statistical analysis; approval of the final version of the manuscript; design and planning of the study; effective participation in research orientation; intellectual participation in the propaedeutic and/or therapeutic conduct of the studied cases; critical review of the literature; critical review of the manuscript.

Flavio Luz: Approval of the final version of the manuscript; design and planning of the study; collection, analysis, and interpretation of data; effective participation in research orientation; intellectual participation in the propaedeutic and/or therapeutic conduct of the studied cases; critical review of the literature; critical review of the manuscript.

## Conflicts of interest

None declared.
